# The Relationship of Serum Macrophage Inhibitory Cytokine – 1 Levels with Gray Matter Volumes in Community-Dwelling Older Individuals

**DOI:** 10.1371/journal.pone.0123399

**Published:** 2015-04-13

**Authors:** Jiyang Jiang, Wei Wen, David A. Brown, John Crawford, Anbupalam Thalamuthu, Evelyn Smith, Samuel N. Breit, Tao Liu, Wanlin Zhu, Henry Brodaty, Bernhard T. Baune, Julian N. Trollor, Perminder S. Sachdev

**Affiliations:** 1 Centre for Healthy Brain Ageing (CHeBA), School of Psychiatry, University of New South Wales, Randwick NSW, Australia; 2 Neuropsychiatric Institute, Prince of Wales Hospital, Randwick NSW, Australia; 3 Centre for Applied Medical Research, St. Vincent’s Hospital and University of New South Wales, Darlinghurst NSW, Australia; 4 School of Biological Science and Medical Engineering, Beihang University, Beijing, China; 5 State Key Laboratory of Cognitive Neuroscience and Learning, Beijing Normal University, Beijing, China; 6 Aged Care Psychiatry, Prince of Wales Hospital, Randwick NSW, Australia; 7 Dementia Collaborative Research Centre, University of New South Wales, Sydney NSW, Australia; 8 Discipline of Psychiatry, School of Medicine, University of Adelaide, Adelaide, SA, Australia; 9 Department of Development Disability Neuropsychiatry, School of Psychiatry, University of New South Wales, Sydney NSW, Australia; Brighton and Sussex Medical School, UNITED KINGDOM

## Abstract

Using circulating inflammatory markers and magnetic resonance imaging (MRI), recent studies have associated inflammation with brain volumetric measures. Macrophage Inhibitory Cytokine–1 (MIC-1/GDF15) is a divergent transforming growth factor – beta (TGF-β) superfamily cytokine. To uncover the underlying mechanisms of the previous finding of a negative association between MIC-1/GDF15 serum levels and cognition, the present study aimed to examine the relationship of circulating MIC-1/GDF15 levels with human brain gray matter (GM) volumes, in a community-dwelling sample aged 70–90 years over two years (Wave 1: n = 506, Wave 2: n = 327), of which the age-related brain atrophy had been previously well defined. T1-weighted MRI scans were obtained at both waves and analyzed using the FMRIB Software Library and FreeSurfer. The results showed significantly negative associations between MIC-1/GDF15 serum levels and both subcortical and cortical GM volumes. GM volumes of the whole brain, cortex, temporal lobe, thalamus and accumbens showed significant mediating effects on the associations between MIC-1/GDF15 serum levels and global cognition scores. Increases in MIC-1/GDF15 serum levels were associated with decreases in cortical and subcortical GM volume over two years. In conclusion, MIC-1/GDF15 serum levels were inversely associated with GM volumes both cross-sectionally and longitudinally.

## Introduction

Inflammation is associated with neuropsychiatric disorders, such as Alzheimer’s disease [1], schizophrenia [2], bipolar disorder [3], and major depressive disorder [4]. It is also a feature of atherosclerosis [5] and stroke [6, 7]. Some recent studies have attempted to better understand the pathogenic mechanisms underlying these associations by examining the relationship between magnetic resonance imaging (MRI) of brain structures and markers of inflammation, such as interleukins (ILs) [8–11]. In addition, there are defined relationships of serum levels of tumor necrosis factor—alpha (TNF-α) with total brain volume [12] and hippocampal volume [13], as well as C-reactive protein (CRP) serum levels with temporal gray matter volume [14] and global and regional white matter integrity measured by fractional anisotropy [15].

A recent study from our group revealed that serum Macrophage Inhibitory Cytokine—1 (MIC-1/GDF15) levels were associated with cognitive decline [16]. The current study, therefore, aimed to determine if this relationship extended to changes in gray matter volume. MIC-1/GDF15 is a divergent member of the transforming growth factor-β (TGF-β) superfamily [17–20]. Its expression is elevated in disease status such as injury [21], malignancy [22–24] and inflammation [19, 25]. Unlike most other inflammatory pathway cytokines regulated by the transcription factor Nuclear Factor-kappa B (NFkB) [26, 27], MIC-1/GDF15 is induced by alternate transcription factors including p53 [28, 29] and Egr-1 [30]. Further, MIC-1/GDF15 is not an acute phase reactant as its serum level is not altered by administration of high doses of corticosteroids [25]. Therefore, it is likely that MIC-1/GDF15 serum levels reflect the activity of cellular processes not sampled by the more commonly studied inflammation associated cytokines.

Whilst MIC-1/GDF15 serum levels are elevated in inflammatory conditions like atherosclerosis [31] and rheumatoid arthritis [25], current evidences from animal models suggest that it may be anti-inflammatory [18, 21, 32], neurotrophic and neuroprotective [20]. Like many other neurotrophic cytokines, MIC-1/GDF-15 mRNA and protein are expressed in the central and peripheral nervous systems throughout fetal development and into adulthood [20, 21]. In adulthood, MIC-1/GDF15 is mainly expressed in the choroid plexus and secreted into cerebrospinal fluid (CSF). Its expression is up-regulated in central nervous system (CNS) injury [21] and intracranial tumors [33], and is elevated in the CSF of patients with CNS tumors and HIV associated neurological disease [18]. MIC-1/GDF15 serum levels have also been related to cognitive decline in the elderly; a recent study from our group identified a strong cross-sectional and prospective negative association between MIC-1/GDF15 serum levels and cognitive function [16]. A MIC-1/GDF15 serum level exceeding 2764 pg/ml was associated with a 20% chance of decline from normal to MCI or dementia.

In our previous study [34], we reported that the age-related brain atrophy over two years, and gender and education impacts on the brain decline in the same sample as the current study. Our findings were similar to those from some benchmark longitudinal studies [35–38]. In this well-defined sample, our current study aimed at investigating the associations between MIC-1/GDF15 serum levels and brain gray matter (GM) volumes, in order to 1) examine the contribution of peripheral inflammation and/or MIC-1/GDF15 itself to the age-related brain decline, and 2) elucidate the mechanisms that may underlie the relationship between MIC-1/GDF15 and cognitive decline. Based on the findings from previous studies, we hypothesize that an elevated level of systemic MIC-1/GDF15 serum levels would correlate with GM atrophy in cortical and subcortical regions, and that the GM volume is a mediator of the previously observed relationships between MIC-1/GDF15 serum levels and cognition.

## Materials and Methods

### Participants

Participants were from the Sydney Memory and Ageing Study (MAS), a community-based longitudinal study recruiting randomly through the electoral roll from two federal government areas in Sydney, New South Wales, Australia. The study background, methods and sample characteristics were reported in details elsewhere [39]. Briefly, 1037 community-dwelling non-demented older individuals aged 70–90 years received neuropsychological tests, medical assessments, and a telephone interview of an informant. The exclusion criteria included dementia, mental retardation, psychotic disorder including schizophrenia or bipolar disorder, multiple sclerosis, motor neuron disease, developmental disability, or progressive malignancy. Individuals with a non-English speaking background to ensure the validity of completing assessments are also disqualified.

The current report involved participants from Wave 1 and Wave 2 of the MAS (two years apart; Table 1). At Wave 1, 506 individuals received both MRI scans and blood tests, and were included in Wave 1 cross-sectional analyses. The number of participants decreased to 327 for Wave 2 due to death or participant drop out. Of the 327 participants, 247 received both MRI scans and blood tests at both waves, and were therefore included in longitudinal analyses.

**Table 1:** Sample characteristics.

	Wave 1	Wave 2
**N (MIC-1/GDF15)**	888	585
**N (MRI)**	551	420
**N (have both MRI and MIC-1)** ^**a**^	506	327
**Age (years) (Mean ± SD)**	78.28 ± 4.59	79.68 ± 4.58
**% Female**	53.6%	51.8%
**Education (years)**	11.80 ± 3.61	11.93 ± 3.71
**Levels of MIC-1/GDF15 (pg/ml) (Mean ± SD)**	1248.48 ± 603.04	1257.62 ± 657.43
**Levels of CRP (mg/L) (Mean ± SD)**	2.90 ± 5.28	2.96 ± 4.69
**Levels of IL-6 (pg/ml) (Mean ± SD)**	6.45 ± 9.58	7.34 ± 15.83
**Apolipoprotein allele ε4**	22.7% (n = 496) ^b^	24.8% (n = 324)
**Cardiovascular disease risk score** ^**c**^ **(Mean ± SD)**	17.24 ± 3.58 (n = 501)	17.07 ± 3.63 (n = 322)
**Stroke**	2.4% (n = 500)	3.1% (n = 322)
**Acute myocardial infarction**	7.4% (n = 503)	6.2% (n = 324)
**Angina**	10.0% (n = 501)	12.5% (n = 319)
**History of transient ischemic attack** ^**d**^	6.3% (n = 495)	7.0% (n = 313)
**History of cancer**	39.9% (n = 504)	42.7% (n = 323)

MIC-1/GDF15 = Macrophage Inhibitory Cytokine—1 / Growth Differentiation Factor 15

CRP = C-Reactive Protein

IL-6 = Interleukin-6

SD = standard deviation

^a^ 247 participants received both MRI scans and blood tests at both Waves.

^b^ The number of subjects varied among different measures due to missing data

^c^ Cardiovascular disease (CVD) risk scores were calculated based on Framingham Heart Disease Risk Score, which was a percentage illustrating the summary of cardiovascular risks of age, sex, systolic blood pressure, use of antihypertensive treatment, cigarette smoking, diabetes mellitus, total cholesterol, high-density lipoprotein (HDL) cholesterol, and body mass index (BMI). The score ranged from 7 to 28 at wave 1, and 7 to 26 at wave 2.

^d^ The data came from self-report

The study was approved by the Human Research Ethics Committees of the University of New South Wales and the South Eastern Sydney and Illawarra Area Health Service. All our participants gave written informed consent. This has been approved by the Institutional Review Board (IRB).

### MIC-1/GDF15 serum levels measurement

The MIC-1/GDF15 serum levels concentration for both Wave 1 and Wave 2 was determined using an enzyme-linked immunosorbent assay (ELISA) as previously described [40]. Briefly, the MIC-1/GDF15 sandwich ELISA was established using the mouse MAb 13C4H3 for antigen capture; and the sheep PAb 233B3-P for detection. Maxisorp 96-well ELISA plates (Nalge Nunc International, Roskilde, Denmark) were coated with MAb 13C4H3 supernatant diluted 1:5 (final Ig concentration was approximately 20 ng/mL) in coating buffer at 4C for 24 h. ELISA plates were then washed three times with 300 μL/well of wash buffer. Nonspecific binding was blocked with 250 μL/well of 1% (wt/vol) BSA in PBS for 2 h at 37 C. rhMIC-1 standard or serum in Ab dil were then added to the plates (100 μL/well) and incubated for 1 h at 37 C. The plates were washed three times, followed by the addition of 100 μL/well of the sheep PAb 233B3-P diluted 1:5000 in Ab dil and incubated for 1 h at 37 C. ELISA plates were then washed three times, and 100 μL/well of biotinylated donkey antisheep IgG diluted to 1:5000 in Ab dil was added and incubated for 1 h at 37 C. The plates were then developed, as for the direct ELISA. The concentration of hMIC-1 in the samples was determined by comparison with the rhMIC-1 standard curve. The standard curve was constructed using standard curve-fitting software supplied with the microplate reader (Pasteur Diagnostics). The level of rhMIC-1 in the standard curve was determined on the basis of a comparison of this standard to a master standard of highly purified recombinant MIC-1. The master standard protein concentration was determined by an average of eight estimations of total amino acid composition. All samples were assayed in triplicate on at least two occasions. Results are presented as the mean ± SD. The CV for all readings was less than 10%.

### Magnetic resonance imaging

276 out of the 506 Wave 1 participants were scanned on a Philips 3T Intera Quasar scanner (Philips Medical Systems, Best, the Netherlands), whereas the remaining 230 Wave 1 and all Wave 2 participants received MRI scans from a Philips 3T Achieva Quasar Dual scanner. Of the 247 participants who received both MRI scans and blood tests at both Wave 1 and 2, 133 individuals were scanned by the first scanner at Wave 1 and then by the second scanner at Wave 2 (i.e. the scans of these people were acquired by the two different scanners at the two time points), whereas the scans of the rest 112 individuals were from the same scanner at both Wave 1 and 2. The two scanners were set to the same parameters for T1-weighted MRI acquisitions: TR = 6.39 ms, TE = 2.9 ms, flip angle = 8°, matrix size = 256 × 256, FOV = 256 × 256 × 190, and slice thickness = 1 mm with no gap in between, yielding 1 × 1 × 1 mm^3^ isotropic voxels.

Since the participants were recruited randomly, little systematic sampling bias was likely to be induced by the change of scanners. At Wave 1, participants underwent scans at the two different scanners did not differ in sex (chi-square value = 0.491, p = 0.483), years of education (p = 0.210), age (p = 0.150), or MIC-1/GDF15 serum levels (p = 0.766). After adjusting age, sex and years of education, we did not observe any GM volume (p = 0.896), white matter (WM) volume (p = 0.892) or total intracranial volume (ICV) (p = 0.451) difference between the participants scanned on the two scanners. To test if scanner type was a moderator of the relationships being investigated, we included into the model the addition of an interaction term consisting of the product of scanner type (coded as a dummy variable) and the independent variable (IV) under investigation (i.e. MIC-1/GDF15 serum levels at Wave 1 or the change in MIC-1/GDF15 serum levels over two years). All p-values for the interaction term were greater than 0.074 and partial R-squares less than 1.26% of the explained variance. We should also point out that, in the regression models for any of these analyses, scanner type, or interaction terms involving this variable, were not confounded with any of the variables (variance inflation factor (VIF) values no greater than 1.649). Therefore, the change of scanners did not affect our results. To adjust for any possible bias introduced by scanners, we coded the two scanners with one dummy variable, i.e. 1 for the scans acquired on the first scanner, and 2 for those from the second scanner. Therefore, 276 out of the 506 participants at Wave 1 were assigned with “1”, and the rest 230 individuals were marked as “2”. This dummy variable was used as a control variable in all analyses that used data from the two scanners (i.e. Wave 1 cross-sectional analyses and all longitudinal analyses).

### Image processing

Freesurfer v5.1.0 (http://surfer.nmr.mgh.harvard.edu/, [41]) was used for extracting global, total cortical, and lobar volumes. Briefly, the cortical model was set up by using intensity normalization, correction and surface deformation. After applying a series of deformable procedures, cortical thickness was then calculated as the closest distance from the gray/white boundary to the gray/CSF boundary at each vertex on the tessellated surface. Cortical volume is calculated as multiplying cortical thickness by cortical surface area.

Subcortical structures were processed by FMRIB Software Library (FSL) v5.0.1 [42]. FSL was used instead of FreeSurfer for subcortical volumes because of a more robust hippocampal segmentation in our preliminary analyses; volumes of 92 manually traced bilateral hippocampi showed stronger correlations with FSL-segmented (r = 0.67 to 0.71) compared to FreeSurfer-segmented (r = 0.51 to 0.52) hippocampal volumes. The correlation coefficient between FSL generated and manually traced hippocampal volume was similar to a previous study of 20 participants in their mid-30s (r = 0.66, [43]). Another advantage for using FSL was its tools conducting vertex-wise surface analyses on subcortical structures, which was used in the current study.

Specifically, non-brain tissue was removed by first registering a standard space brain template to the individual brain image, and then running an automated skull stripping procedure based on the SPM5 skull-cleanup tool [44] to obtain the brain. FMRIB’s Integrated Registration and Segmentation Tool (FIRST v4.1) [45], was then applied to generate 15 subcortical structures (7 on each hemisphere and brainstem). The FIRST algorithm modeled each participant's subcortical structure as a surface mesh, using a Bayesian model incorporating a training set of all images. The boundary of the structure was determined by a correction procedure, using a Gaussian mixture-model and Markov Random field (for more details, refer to [45]). To reduce the number of comparisons, the volume of structures on the left and right hemisphere were combined.

### Quality control

The quality of FSL outcomes was controlled by applying ENIGMA protocols (http://enigma.ini.usc.edu/protocols/imaging-protocols/). Briefly, skull stripping was conducted before running FIRST to ensure the registration accuracy. After running FIRST, registration was checked for accuracy; an outline of the templates was projected onto the slices extracted from each of the coronal, sagittal and axial planes of each linearly transformed scan. We confirmed that the size and orientation of the individual brain corresponded with the template, and the lobes were correctly located. For each brain, the volume of each subcortical structure was then extracted. Segmentations with the volume exceeding mean±1.96SD were then checked for whether the labels accurately cover the full subcortical structures using the FSLVIEW toolbox. FreeSurfer results were also visually checked for segmentation and registration accuracy using TKMEDIT toolbox. Scans were excluded if they failed visual quality control. The number of excluded scans for each structure is shown in S1 Table (in S1_Tables.docx).

### Global cognition scores (GCSs)

In MAS, we recruited six cognition domains to evaluate the participants’ neuropsychological performances: 1) *processing speed* was assessed by the Wechsler Adult Intelligence Scale-III Digit Symbol-Coding [46] and Trail Making Test part A (TMT A) [47]; 2) *memory* was evaluated by Logical Memory Story A (delayed recall) [48], Rey Auditory Verbal Learning Test (RAVLT) [47], and Benton Visual Retention Test recognition (BVRT) [49]; 3) *verbal memory* used the same measures in the memory domain except for BVRT; 4) *language* was tested by Boston Naming Test (30 items) [50] and Semantic Fluency Test (animals) [47]; 5) *visuo-spatial* ability was from Block Design frim the Wechsler Adult Intelligence Scale—Revised [51]; 6) *executive function* was evaluated by Controlled Oral Word Association Test (FAS) and Trail Making Test part B (TMT B) [47].

Raw scores were standardized (i.e. converted to Z-scores), based on the means and SDs of a normal cognition reference group derived from the cohort. Domain scores were calculated by averaging the Z-scores of the component tests. GCSs were obtained by averaging domain Z-scores.

### Statistical analyses

In order to examine the associations between MIC-1/GDF15 serum levels and brain structures on a vertex basis, we carried out shape analyses on both cortical and subcortical structures using toolboxes from FreeSurfer and FSL.

Subcortical surface analyses were performed by using the vertex analysis function provided by FMRIB’s Integrated Registration Segmentation Toolkit (FIRST). Briefly, after segmenting the subcortical structures, the deformable surfaces of deep GM structures were used to automatically parameterize the volumetric labels in terms of meshes. The normalized intensities along the surface of meshes were sampled and modeled. The shape and appearance model was based on multivariate Gaussian assumptions. Shape was then expressed as a mean with modes of variation (principal components). The results were considered to be statistically significant at areas with p < 0.05, after correcting for multiple comparisons using false discovery rate (FDR) correction.

We conducted cortical shape analysis using QDEC tool box provided by FreeSurfer (www.surfer.nmr.mgh.harvard.edu), in which general linear models were applied on a vertex basis. The results were projected onto the template, which were then corrected for multiple comparisons using FDR at a level of 0.05.

The region of interest (ROI) analyses were performed using SPSS v21.0.0 (IBM Corp. Released 2012. IBM SPSS Statistics for Windows, Version 21.0. Armonk, NY: IBM Corp). The distribution of MIC-1/GDF15 serum levels and GM volumes were examined for normality. Outliers were removed by applying a cut-off of ±3 standard deviations (SDs), i.e. any value exceeded ±3SD was considered as an outlier, and therefore excluded.

To assess the cross-sectional relationships at each Wave between MIC-1/GDF15 serum levels and brain GM volumes, two sets of multiple linear regression models were fitted. The first model adjusted for age, sex, years of education, ICV, as well as scanner type when Wave 1 volumetric measures were used (model 1). The second model included additional control variables selected from a set of potential confounding variables. The possible confounders include history of cerebrovascular accident (CVA), transient ischemic attack (TIA), acute myocardial infarction (AMI), angina, cancer, APOE4 genotype, race, cardiovascular disease (CVD) risk scores, C-reactive protein (CRP) plasma levels, and interleukin (IL)– 6 serum levels. The selection of these additional control variables was done using the stepwise procedure for model reduction, with the p values for variable entry and removal being set at 0.05 and 0.10, respectively. Such regression analyses were also performed to examine the extent to which MIC-1/GDF15 serum levels at Wave 1 predicted changes in GM volumes from Wave 1 to Wave 2, as well as whether Wave 1 GM volumes predicted changes in MIC-1/GDF15 serum levels over two years, and a further set of analyses were carried out to investigate the relationships between changes in MIC-1/GDF15 serum levels from Wave 1 to Wave 2 with the corresponding changes in GM volumes across the two Waves. Using the same covariates and settings, a regression analysis was also carried out to investigate whether the association between changes in MIC-1/GDF15 and in GM volumes depends on the initial levels of MIC-1/GDF15, that is whether Wave 1 MIC-1/GDF15 is a moderator of this relationship. This was done by including in the model MIC-1 baseline levels as well as the interaction product term between these levels and the change in MIC-1 levels.

The associations between MIC-1/GDF15 serum levels and GCSs were also examined in two models. In the first model, we controlled for demographic factors, including age, sex and years of education. The second model further included other possible confounders, including history of CVA, TIA, AMI, angina, cancer, CVD risk scores, CRP plasma levels and IL-6 serum levels. The second model used stepwise procedure for model reduction. The possible mediation effects of the GM ROIs on the relationships of MIC-1/GDF15 serum levels with GCSs were investigated using Sobel tests.

The analyses were corrected using FDR at a level of 0.05. Possible non-linear effects were examined by the inclusion of an additional MIC-1/GDF15 serum levels-squared term in the regression models. For these analyses, the MIC-1/GDF15 serum levels were centered, to avoid multicollinearity between itself and its squared value.

## Results

### Demographic characteristics

Table 1 presents the demographic characteristics and medical conditions of the study participants. While all participants at Wave 1 were non-demented, 10 were diagnosed with dementia at Wave 2. MIC-1/GDF15 serum levels at Wave 1 and Wave 2 were significantly correlated (n = 315, r = 0.812, p < 0.001), indicating a good test-retest reliability.

### The associations between MIC-1/GDF15 serum levels and brain GM volumes—cross-sectional analyses

Table 2 summarizes the associations between MIC-1/GDF15 serum levels and brain GM volumes at both Wave 1 and 2. In general, MIC-1/GDF15 serum levels showed negative relationships with brain GM volumes. The effects were more consistent and significant for Wave 1 data compared to Wave 2. The difference in the strength of the results could be due in part to non-random attrition. Logistic regression analysis showed a statistically significant prediction by MIC-1 levels at Wave 1 of who dropped out of the study by Wave 2 (p = 0.025). Since the MIC-1/GDF15 serum levels at the two waves are highly correlated (r = 0.812, p < 0.001), this would tend to remove from the Wave 2 sample individuals who would otherwise have relatively higher concentrations of MIC-1/GDF15 and so reduce the correlations with brain GM volumes at Wave 2. However, despite being statistically significant, the strength of this prediction was small (odds ratio = 1.177, Z-scores of Wave 1 MIC-1/GDF1 levels were used to avoid the influence of the unit of measurement of Wave 1 MIC-1/GDF15). So whether this could contribute to explaining the difference between the two waves in the strength of associations is not clear.

**Table 2:** Regression analyses for the relationships of MIC-1/GDF15 level with brain GM volume at both wave 1 and 2.

		Wave 1				Wave 2			
		Model 1		Model 2		Model 1		Model 2	
		β	p	β	p	β	p	β	p
**Whole brain GM**		-.135	.000*	-.132	.000*	-.077	.052	-.075	.056
**Cortices**	**Total cortical GM**	-.115	.001*	-.112	.001*	-.060	.131	-.049	.217
	**Frontal**	-.096	.007*	-.093	.008*	-.034	.453	-.022	.628
	**Parietal**	-.116	.001*	-.113	.001*	-.066	.120	-.054	.207
	**Temporal**	-.134	.000*	-.131	.000*	-.077	.078	-.077	.078
	**Occipital**	-.090	.034*	-.087	.038*	-.018	.716	-.018	.716
	**Insula**	-.064	.031*	-.064	.031*	-.101	.009	-.091	.018
**Subcortical structures**	**Total subcortical GM**	-.120	.003*	-.117	.004*	-.103	.047	-.099	.050
	**Hippocampus**	-.127	.008*	-.127	.008*	-.111	.062	-.111	.062
	**Thalamus**	-.180	.000*	-.177	.000*	-.114	.054	-.110	.057
	**Caudate**	-.023	.604	-.023	.604	.003	.961	.003	.961
	**Putamen**	-.042	.364	-.040	.380	-.022	.722	-.022	.722
	**Pallidum**	-.140	.007*	-.152	.003*	-.054	.409	-.041	.507
	**Amygdala**	-.051	.323	-.048	.353	-.077	.233	-.077	.233
	**Accumbens**	-.209	.000*	-.209	.000*	-.111	.075	-.106	.081
	**Brainstem**	-.124	.002*	-.124	.002*	-.074	.155	-.062	.225

* statistically significant after corrected for multiple comparisons using False Discovery Rate (FDR) at level of 0.05

Model 1: adjusting for age, sex, years of education, scanner (for wave 1 analyses), and intracranial volume (ICV)

Model 2: adjusting for confounders after model reduction using stepwise procedure. The full initial confounder list includes history of cerebrovascular accident (CVA), transient ischemic attack (TIA), acute myocardial infarction (AMI), angina, cancer, APOE4 genotype, race, cardiovascular (CVD) risk scores, C-reactive protein (CRP) level, and interleukin (IL)– 6 level.

**Fig 1:**
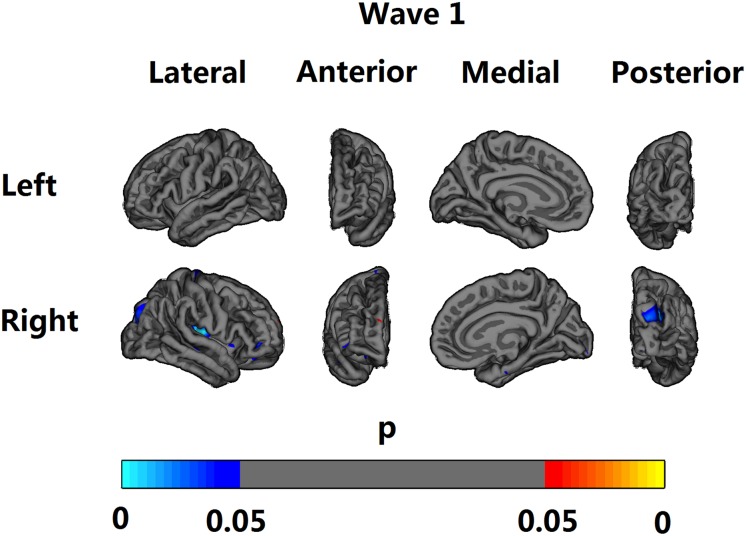
Vertex-based analyses of associations between Macrophage Inhibitory Cytokine—1 (MIC-1/GDF15) serum levels and cortical volume at Wave 1. The result was adjusted for age, sex, years of education, scanner, and intracranial volume (ICV) (Model 1). The results were corrected at a false discovery rate (FDR) of 0.05 and projected onto a semi-inflated brain. Negative correlations are indicated by cyan and blue, and positive correlations by red and yellow.

**Fig 2:**
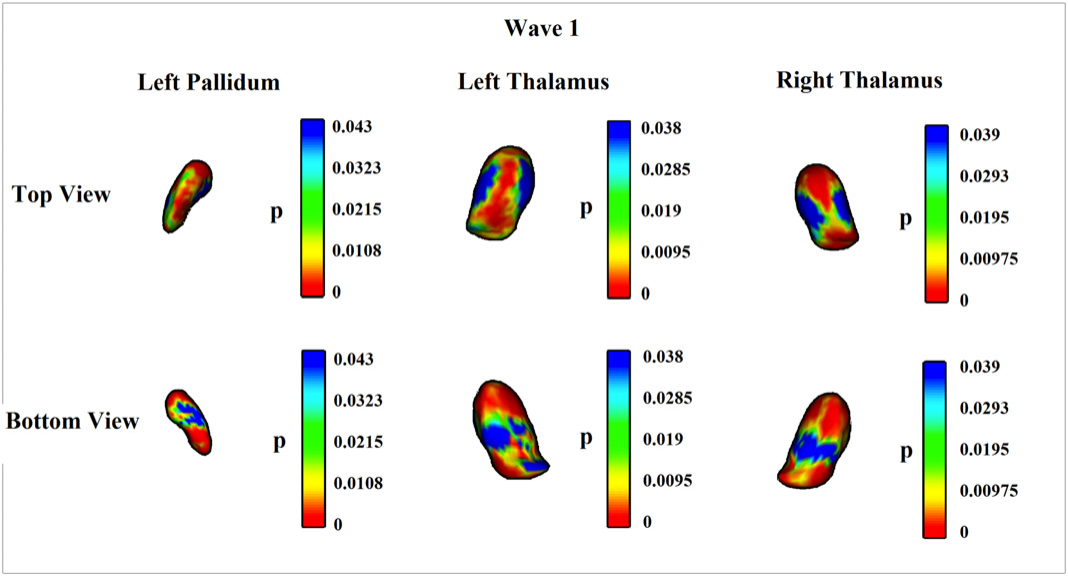
Vertex-wise analyses of the relationships between MIC-1/GDF15 serum levels and subcortical structures at Wave 1. The result was after FDR correction and adjusted for age, sex, years of education, scanner, and intracranial volume (ICV) (Model 1). Regions showing statistical significance were negatively correlated with MIC-1/GDF15 serum levels. Left pallidum and bilateral thalami survived FDR correction. After FDR correction, the ranges of statistical significance (p values) were changed to 0–0.043 for left pallidum, 0–0.038 for left thalamus, and 0–0.039 for right thalamus.

At Wave 1, after FDR correction, all tested ROIs except caudate, putamen and amygdala showed statistically significant inverse relationships with MIC-1/GDF15 serum levels. Cortical voxel-based analyses supported this by showing, after FDR correction, significant inverse relationships of MIC-1/GDF15 serum levels with the following regions: transverse temporal and superior parietal, as well as some parts of precentral, insula, pars trangularis and pars orbitalis regions on the right hemisphere (Fig 1). After FDR correction, subcortical vertex-based shape analyses also showed a similar pattern as ROI analyses; MIC-1/GDF15 serum levels negatively correlated with most of the left pallidum and bilateral thalami surfaces (Fig 2). At Wave 2, MIC-1/GDF15 serum levels were significantly inversely associated with total subcortical and insula GM volumes, but the results did not survive FDR correction. The negative associations between MIC-1/GDF15 and whole brain, temporal, hippocampus, thalamus, and accumbens GM volumes, also showed trends towards statistical significance. The cortical and subcortical voxel-wise analyses did not reach statistical significance at any region at Wave 2 after FDR correction. None of the ROIs showed a non-linear relationship with MIC-1/GDF15 serum levels at either Wave 1 or Wave 2.

### The associations between MIC-1/GDF15 serum levels and brain GM volumes—longitudinal analyses

Using data from Wave 1 and Wave 2, we first investigated whether MIC-1/GDF15 serum levels at Wave 1 could predict the GM changes over two years (Table 3). Generally, there was a weak non-significant trend that a higher MIC-1/GDF15 serum level at Wave 1 was associated with less cortical and subcortical GM atrophy over two years. The only association reached statistical significance was between Wave 1 MIC-1/GDF15 serum levels and changes in insula volumes; individuals with higher initial MIC-1/GDF15 tended to have greater insula atrophy in the following two years. To further examine if higher initial MIC-1/GDF15 serum levels have protective effects on GM, we categorized both GM volumes (divided by ICV) and MIC-1/GDF15 serum levels at Wave 1 into high and low groups, but did not observe significantly less 2-year GM atrophy in the participants with higher MIC-1/GDF15 serum levels and more preserved GM volumes at Wave 1, compared to the ones with lower MIC-1/GDF15 serum levels and smaller GM volumes, after FDR correction.

**Table 3:** Regression analyses for the relationships of MIC-1/GDF15 at wave 1 with the changes in brain GM volume over two years.

		Model 1		Model 2	
		β	p	β	p
**Whole brain GM**		.110	.117	.114	.102
**Cortices**	**Total cortical GM**	.106	.122	.110	.102
	**Frontal**	.079	.251	.082	.230
	**Temporal**	.061	.400	.064	.375
	**Parietal**	.109	.117	.113	.097
	**Occipital**	.043	.551	.043	.551
	**Insula**	-.161	.028	-.156	.031
**Subcortical structures**	**Total subcortical GM**	.061	.482	.073	.389
	**Hippocampus**	.036	.635	.042	.573
	**Thalamus**	.046	.539	.046	.539
	**Caudate**	.079	.276	.079	.276
	**Putamen**	-.013	.867	-.013	.867
	**Pallidum**	.057	.456	.057	.456
	**Amygdala**	-.015	.852	-.015	.852
	**Accumbens**	-.063	.403	-.063	.403
	**Brainstem**	.058	.439	.063	.392

Model 1: adjusting for age, sex, years of education, scanner, and ICV

Model 2: adjusting for confounders after model reduction using stepwise procedure. The full confounder list includes history of CVA, TIA, AMI, angina, cancer, APOE4 genotype, race, CVD risk scores, CRP level, and IL-6 level.

Since the causality between MIC-1/GDF15 and brain atrophy was uncertain, we also tested the possibility of Wave 1 GM volumes predicting changes in MIC-1/GDF15 serum levels over two years (Table 4). The changes in MIC-1/GDF15 serum levels over two years were not significantly associated with the volumes of any tested GM regions at Wave 1.

**Table 4:** Regression analyses for the associations between Wave 1 GM volumes and changes in MIC-1/GDF15 serum levels.

	Model 1		Model 2	
	Beta	P	Beta	P
Whole brain	.005	.946	.001	.989
Total cortical	.034	.616	.030	.649
Frontal	.016	.797	.009	.890
Parietal	.026	.688	.029	.654
Temporal	.023	.722	.025	.699
Occipital	.055	.404	.048	.461
Insula	-.022	.715	-.016	.792
Total subcortical	.028	.688	.028	.688
Hippocampus	.015	.825	.014	.840
Thalamus	.105	.117	.088	.189
Caudate	.004	.951	.004	.951
Putamen	.015	.816	.015	.816
Pallidum	.085	.174	.061	.330
Amygdala	.033	.581	.009	.876
Accumbens	.116	.075	.116	.075
Brainstem	.066	.274	.065	.283

Model 1: adjusting for age, sex, years of education, scanner, and ICV

Model 2: adjusting for confounders after model reduction using stepwise procedure. The full confounder list includes history of CVA, TIA, AMI, angina, cancer, APOE4 genotype, race, CVD risk scores, CRP level, and IL-6 level.

**Fig 3:**
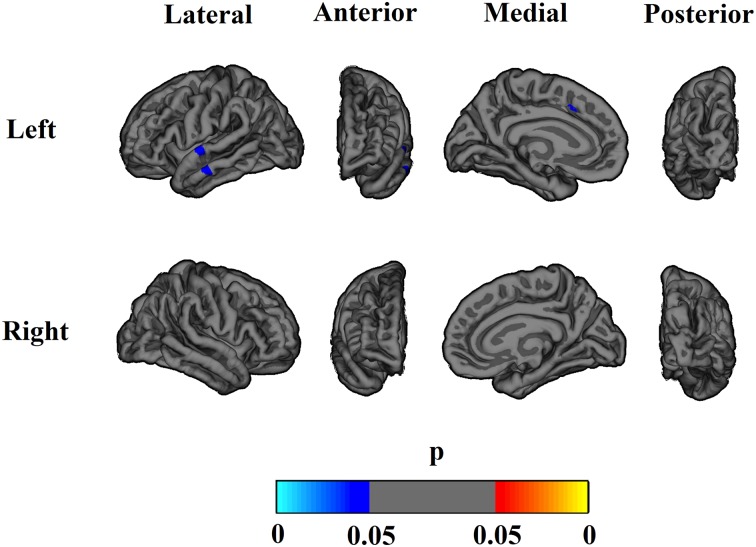
Associations of MIC-1/GDF15 level changes and cortical volume changes over two years. The result was adjusted for age, sex, years of education, scanner, and ICV (Model 1). Negative correlations are indicated by cyan and blue, and positive correlations by red and yellow. The results were after correction for multiple comparisons using FDR. Volume changes of parts of superior temporal, middle temporal and superior frontal regions negatively correlated with MIC-1/GDF15 level change over two years.

The associations of MIC-1/GDF15 serum level changes with GM volume changes are presented in Table 5 and Fig 3. GM volumetric changes of all tested GM structures, except occipital lobe, insula, caudate, putamen, pallidum, accumbens and brainstem, showed significantly (p < 0.05) or marginally (p ≤ 0.061) negative relationships with the changes in MIC-1/GDF15 serum levels. After FDR correction, changes in parietal lobe, total cortical, total subcortical, and whole brain GM volumes still showed significant inverse relationships with changes in MIC-1/GDF15 serum levels, i.e. an increase in MIC-1/GDF15 serum levels was associated with a decrease of GM volumes. No statistically significant non-linear effects were observed for the relationships of changes in MIC-1/GDF15 serum levels with changes in any of the GM volumes.

**Table 5:** Regression analyses for the relationships of MIC-1/GDF15 changes with brain GM volume changes over two years.

		Model 1		Model 2	
		Beta	p	Beta	p
**Whole brain GM**		-.235	.000*	-.226	.000*
**Cortices**	**Total cortical GM**	-.173	.007*	-.165	.008*
	**Frontal**	-.130	.042	-.124	.051
	**Temporal**	-.146	.031	-.136	.042
	**Parietal**	-.171	.008*	-.161	.011*
	**Occipital**	-.084	.216	-.084	.216
	**Insula**	-.088	.198	-.099	.140
**Subcortical structures**	**Total subcortical GM**	-.261	.001*	-.234	.002*
	**Hippocampus**	-.138	.048	-.130	.061
	**Thalamus**	-.149	.031	-.149	.031
	**Caudate**	-.074	.271	-.074	.271
	**Putamen**	-.099	.159	-.099	.159
	**Pallidum**	-.047	.515	-.047	.515
	**Amygdala**	-.140	.052	-.140	.052
	**Accumbens**	-.106	.129	-.106	.129
	**Brainstem**	-.057	.413	-.049	.478

* statistically significant after corrected for multiple comparisons using FDR at level of 0.05

Model 1: adjusting for age, sex, years of education, scanner, and ICV

Model 2: adjusting for confounders after model reduction using stepwise procedure. The full confounder list includes history of CVA, TIA, AMI, angina, cancer, APOE4 genotype, race, CVD risk scores, CRP level change, and IL-6 level change.

**Fig 4:**
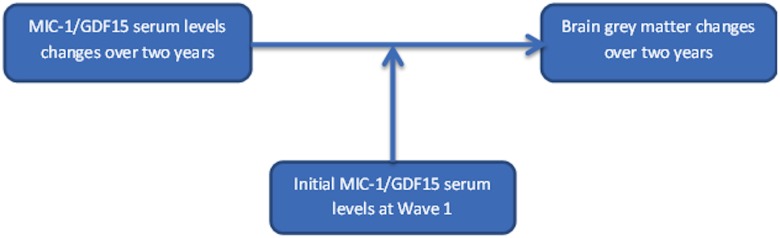
The moderation analysis model. A graphical illustration of the moderation analysis of whether the initial MIC-1/GDF15 serum levels at Wave 1 moderated the strength of the associations between changes in MIC-1/GDF15 serum levels and GM volumes.

To examine if the initial MIC-1/GDF15 serum levels at Wave 1 was a moderator of the strength of the associations between MIC-1/GDF15 serum levels changes and GM changes (the model is shown in Fig 4), we carried out further regression analyses with the Wave 1 MIC-1/GDF15 serum levels and the interaction between MIC-1/GDF15 serum levels at Wave 1 and changes in MIC-1/GDF15 serum levels over two years, also being included in the model. The results showed that wave 1 MIC-1/GDF15 serum levels was not a moderator of the associations of changes in MIC-1/GDF15 serum levels with GM volumetric changes over two years.

We did not control for cognition (e.g. Mild Cognitive Impairment (MCI) and Alzheimer’s Disease (AD)) in our statistical analyses, because we consider cognition changes as an outcome rather than a cause of brain volume changes. However, there is a possibility that some biochemical conditions associating with the conversion to AD and MCI has common influences on both MIC-1/GDF15 serum levels and brain volumes. To test this possibility, we repeated all analyses in a normal ageing sample (excluding MCI and AD participants from the full sample) with the same covariates described in Section 2.6. In both cross-sectional and longitudinal analyses, the majority of statistically significant associations reported for the full sample were repeated in the analyses of normal ageing participants (S2 Table in S2_Table.docx, S3 Table in S3_Table.docx, and S4 Table in S4_Table.docx). The results removed the possibility that our findings could be accounted for by those converting to MCI/AD.

### The associations between MIC-1/GDF15 serum levels and GCSs

Using the cognition data, we first tested the relationships between MIC-1/GDF15 serum levels and the GCSs. Cross-sectionally, MIC-1/GDF15 serum levels were negatively associated with the GCS at both Waves (at Wave 1, Model 1: β = -.096, p = 0.038, Model 2: β = -0.101, p = 0.029; at Wave 2, Model 1:β = -0.152, p = 0.011, Model 2: β = -0.134, p = 0.023). Prospectively, the relationship between Wave 1 MIC-1/GDF15 and Wave 2 GCS (controlling for Wave 1 GCS in addition to other control variables) was not statistically significant (Model 1: β = -0.049, p = 0.194; Model 2: β = -0.045, p = 0.226). Moreover, changes in MIC-1/GDF15 serum levels over two years was not significantly associated with Wave 2 GCS, after controlling for Wave 1 GCS (Model 1: β = 0.000, p = 0.991; Model 2: β = -0.003, p = 0.922). The results were in line with our previous study [16]. Some slight differences in the strength of associations were possibly due to the significantly smaller sample size in the current study because of excluding the participants who did not have MRI scans.

### The mediation effects of GM volumes on the associations between MIC-1/GDF15 serum levels and GCSs

Using the Sobel tests, we then tested the possible mediation effects of GM volumes on the statistically significant relationships between MIC-1/GDF15 and GCSs. Since the mediation effects would not exist if the relationships between the independent variable (MIC-1/GDF15 serum levels in our case) and the possible mediator (GM volumes) did not reach statistical significance, we only tested the potential mediation effects for the GM regions with statistically significant associations with MIC-1/GDF15 (Table 6). GM volumes of the whole brain, cortex, temporal lobe, thalamus and accumbens showed significant mediating effects on the associations between MIC-1/GDF15 serum levels and GCSs at Wave 1. At Wave 2, insula and total subcortical GM volumes showed significant relationships with MIC-1/GDF15, but were not mediators for the associations between MIC-1/GDF15 and GCS.

**Table 6:** Sobel tests for the mediation effects of the GM measures on the statistically significant relationships between MIC-1/GDF15 and GCS at Wave 1 and 2.

		Coeff1	SE1	Coeff2	SE2	Sobel	SE3	p
Whole brain GM (Wave 1)	Model 1	-14.631	3.468	3.957E-6	1E-6	-2.886	2.006E-5	.004
	Model 2	-14.348	3.438	3.957E-6	1E-6	-2.871	1.977E-5	.004
Total cortical GM (Wave 1)	Model 1	-9.712	2.800	5.097E-6	2E-6	-2.054	2.410E-5	.040
	Model 2	-9.450	2.768	5.097E-6	2E-6	-2.042	2.359E-5	.041
Frontal GM (Wave 1)	Model 1	-3.132	1.153	1.059E-5	5E-6	-1.670	1.986E-5	.095
	Model 2	-3.044	1.148	1.059E-5	5E-6	-1.655	1.948E-5	.098
Parietal GM (Wave 1)	Model 1	-2.962	.906	9.330E-6	6E-6	-1.404	1.968E-5	.160
	Model 2	-2.889	.899	9.330E-6	6E-6	-1.400	1.926E-5	.162
Temporal GM (Wave 1)	Model 1	-2.838	.765	1.725E-5	7E-6	-2.053	2.385E-5	.040
	Model 2	-2.760	.754	1.725E-5	7E-6	-2.044	2.329E-5	.041
Occipital GM (Wave 1)	Model 1	-.930	.436	1.930E-5	1.2E-5	-1.284	1.398E-5	.199
	Model 2	-.903	.434	1.930E-5	1.2E-5	-1.272	1.370E-5	.203
Insula GM (Wave 1)	Model 1	-.226	.105	2.933E-5	4.8E-5	-.588	1.128E-5	.557
	Model 2	-.226	.105	3.033E-5	4.8E-5	-.606	1.131E-5	.544
Total subcortical GM (Wave 1)	Model 1	-1.497	.501	2.209E-5	1.1E-5	-1.667	1.984E-5	.096
	Model 2	-1.454	.499	2.209E-5	1.1E-5	-1.654	1.942E-5	.098
Hippocampus GM (Wave 1)	Model 1	-.228	.085	1.17E-4	6E-5	-1.577	1.691E-5	.115
	Model 2	-.228	.085	1.17E-4	6E-5	-1.577	1.691E-5	.115
Thalamus GM (Wave 1)	Model 1	-.517	.123	1.91E-4	4E-5	-3.155	3.130E-5	.002
	Model 2	-.508	.122	1.90E-4	4E-5	-3.131	3.083E-5	.002
Pallidum GM (Wave 1)	Model 1	-.172	.063	9.283E-5	8.1E-5	-1.057	1.511E-5	.291
	Model 2	-.187	.063	9.283E-5	8.1E-5	-1.069	1.624E-5	.285
Accumbens GM (Wave 1)	Model 1	-.081	.019	.001	2.66E-4	-2.820	2.873E-5	.005
	Model 2	-.081	.019	.001	2.65E-4	-2.826	2.867E-5	.005
Brainstem GM (Wave 1)	Model 1	-.724	.229	1.838E-5	2.2E-5	-.808	1.647E-5	.419
	Model 2	-.724	.229	1.831E-5	2.2E-5	-.805	1.647E-5	.421
Insula GM (Wave 2)	Model 1	-.320	.121	3.075E-5	6.1E-5	-.495	1.987E-5	.620
	Model 2	-.288	.121	2.847E-5	6.0E-5	-.465	1.762E-5	.642
Total subcortical GM (Wave 2)	Model 1	-1.010	.507	4.253E-5	1.4E-5	-1.666	2.579E-5	.096
	Model 2	-.974	.496	3.514E-5	1.5E-5	-1.505	2.274E-5	.132

Coeff1: raw (unstandardized) regression coefficient for the association between MIC-1/GDF15 and GM volumes

SE1: standard error of Coeff1

Coeff2: raw (unstandardized) coefficient for the association between GM volumes and GCSs (the MIC-1/GDF15 serum level is also included in the model as an independent variable)

SE2: standard error of Coeff2

Sobel: Sobel test statistic

SE3: standard error of the Sobel test statistic

p: p-value for the Sobel test

## Discussion

To the best of our knowledge, this is the first study to investigate the relationship between the MIC-1/GDF15 serum levels and human brain GM volumes, using both cross-sectional and longitudinal data. After correction for multiple comparisons, GM volumes of most subcortical and cortical regions examined, with the exception of caudate, putamen and amygdala, were negatively correlated with MIC-1/GDF15 serum levels at the initiation of the study period (Wave 1). Over the course of the ensuing two years, after FDR correction, the changes in parietal lobe, total cortical, total subcortical, and whole brain GM volumes were significantly negatively correlated with MIC-1/GDF15 serum level changes. Individuals with higher Wave 1 MIC-1/GDF15 serum levels tend to have less GM volume shrinkage over two years. However, none of the associations between Wave 1 MIC-1/GDF15 serum levels and GM volumetric changes over two years reached statistical significance. The negative relationship of MIC-1/GDF15 serum levels with brain GM volume is consistent with our previous study demonstrating a negative relationship between cognition and MIC-1/GDF15 serum levels [16], and those of other groups investigating the associations of MIC-1/GDF15 with all-cause mortality [52, 53]. We also revealed that GM loss was one of the mechanisms for the linkage between elevated MIC-1/GDF15 serum levels and cognitive degeneration [16].

In our previous longitudinal study [34], we examined 2-year atrophy of cortical and subcortical structures in the same cohort as the current study, though there were some small variations on the exact number of scans in each wave due to the availability of particular parameters and measures. The results showed significant and widespread brain atrophy in two years. The atrophy pattern is similar to previous benchmark longitudinal studies [35–38]. Therefore, the current study investigated the contribution of MIC-1/GDF15 to brain volumes based on a sample with valid brain atrophy patterns.

MIC-1/GDF15 is widely distributed within the CNS and is present in the cerebrospinal fluid (CSF). Its mRNA and protein have been detected in a variety of brain regions, including cortex, hippocampus, striatum, pons, and medulla oblongata [20], making it plausible that it has an influence on brain structures. In adult rats, the protective effects of MIC-1/GDF15 on 6-hydroxydopamine (6-OHDA) lesioned nigrostriatal dopaminergic neurons lasted for at least one month [20], which indicates that its effects on the brain may be relatively long lasting.

MIC-1/GDF15 has antithetical properties which fuel the debate whether it is beneficial or harmful [54]. It inhibits the hypokalemia-induced neuron apoptosis [55], but promotes apoptosis of cancer cells [56]. It stimulates vessel development in the context of melanomas [57], but suppressed angiogenesis in the Matrigel plug assay *in vivo* [58]. Our finding may indicate that the effects of MIC-1/GDF15 are complex. Higher serum levels of MIC-1/GDF15 appear to be associated with some protection against declining GM volume in the future. This finding suggests that MIC-1/GDF15 acts as a protective factor. However, higher serum MIC-1/GDF15 levels at Wave 1 were clearly associated with reduced GM volumes. An alternative explanation is that similar to IL-6, MIC-1/GDF15 might have both pro- and anti-inflammatory properties in the CNS. Previous studies have found that IL-6, which has both pro- and anti-inflammatory properties [59, 60], was negatively correlated with brain volumetric measures [10]. Our finding that the neurotrophic effects of MIC-1/GDF15 might not be able to compensate for CNS injury may be due to the trophic effects of MIC-1/GDF15 acting on existing neurons rather than promoting their numeric expansion [20].

The observed effect of MIC-1/GDF15 on the CNS could also be due to its impact on the cerebrovascular system. MIC-1/GDF15 has been associated with cardiovascular events [31], in which atherosclerosis is the most common underlying pathological mechanism. De Jager et al. [61] reported that removing the gene for MIC-1/GDF15 leads to an increase in atherosclerotic plaque stability, suggesting that the presence of MIC-1/GDF15 may promote plaque rupture and consequent thromboembolic or occlusive events that directly impact the CNS. A previous study had associated atherosclerosis with brain GM atrophy [14], thereby supporting this mechanism of action.

Previous studies showed that MIC-1/GDF15 may serve to inhibit the late phase of macrophage activation [17, 19]. In addition, Johnen et. al. found that MIC-1/GDF15 was likely to be produced by atherosclerostic lesions in an attempt to limit or repair the lesions [32]. This may shed light on the finding that although observing clearly negative associations between MIC-1/GDF15 serum levels and GM volume at Wave 1 and longitudinally, we found a trend that a higher MIC-1/GDF15 serum level at Wave 1 was associated with less cortical and subcortical shrinkage in the ensuing two years.

Although statistically significant negative associations were observed between MIC-1/GDF15 serum levels and brain GM volumes in the current study, we were unable to conclude whether MIC-1/GDF15 has direct effects on brain. MIC-1/GDF15 may be a marker of brain damage due to other reasons, perhaps as a homeostatic response of the body and brain to such damage which in itself is not involved in pathogeneses, or not sufficient to contain or ameliorate the damage. Like other members from TGF-β superfamily, MIC-1/GDF15 is understood to be up-regulated in the lesion process. In a study of adult rat brains, Schober et al. [21] reported detectable MIC-1/GDF15 mRNA six hours after cryogenic lesion of the cortex, however no data is available for humans. The negative correlation between MIC-1/GDF15 serum levels and GM volume in the current report may provide a clue to an analogous result as in animal models. As a result, the inverse relationship may be due to the fact that GM shrinkage induced by ageing or neurodegenerative process [34] is perhaps the cause of the elevation of MIC-1/GDF15 serum levels. This was supported by our finding that MIC-1/GDF15 serum level is a marker but not predictor of cortical change over two years.

Future longitudinal studies with multiple time points are needed to examine the presence or absence of a causal relationship between MIC-1/GDF15 serum level change and brain volumetric change in humans. Since the shape analysis showed a relatively widespread association of bilateral thalami with MIC-1/GDF15 serum levels, which was also supported by our ROI analyses, further investigations on white matter (WM) fibers, especially on thalamo-cortico-thalamic circuits, is worthy of further examination.

Since the number of participants was lower at Wave 2, the statistical power was limited for the analyses using Wave 2 data. Despite the lack of statistical significance, all Wave 2 results were consistently in the same direction as the corresponding Wave 1 findings. The follow-up of the participants in the present study was only for two years, and it is possible that a longer follow-up is necessary to examine the predictive ability of MIC-1/GDF15 serum levels as has been seen in other studies [62]. It is also possible that there is a real but small relationship between initial MIC-1/GDF15 serum levels and changes in brain GM volumes, but that these were not shown to be statistically significant due to a lack of power. A power analysis shows that our sample size would be adequate to reliably detect relatively small effects sizes of beta greater than about 0.18, with power of 0.8 and alpha rate of 0.05. Larger samples would be required, to reliably detect real effect sizes smaller than this value. Including CSF levels of MIC-1/GDF15 in the analyses would be helpful. There were too few dementia patients in our sample to determine what the serum or CSF levels of MIC-1/GDF15 are once dementia develops.

Although we found statistically significant associations between MIC-1/GDF15 serum levels and GM volumes, a relatively small portion (no more than 5.2%) of GM volumes and their changes over time can be explained by MIC-1/GDF15 serum levels and their changes in our sample (S5 Table in S5 Table.docx, S6 Table in S6 Table.docx, and S7 Table in S7 Table.docx). Therefore, the clinical utility of this cytokine as a marker of age-related brain decline needs further investigations.

In conclusion, the present study found an inverse relationship between MIC-1/GDF15 serum levels and brain GM volumes both cross-sectionally and longitudinally. The GM decline was one of the reasons for the negative associations between MIC-1/GDF15 serum levels and cognition.

## Supporting Information

S1 Table Numbers of scans excluded for each structure after quality control (DOCX)

S2 Table The association between MIC-1/GDF15 serum levels and brain GM volumes at Wave 1 and 2 in normal ageing participants (DOCX)

S3 Table Regression analyses for the relationships of MIC-1/GDF15 at wave 1 with the changes in brain GM volume in normal ageing participants over two years (DOCX)

S4 Table The association between MIC-1/GDF15 serum level changes and brain GM volumetric changes in two years in normal ageing participants (DOCX)

S5 Table The R-square change after involving MIC-1/GDF15 serum levels in the associations between MIC-1/GDF15 and brain GM volumes, controlling for all other covariates, at Wave 1 and 2 (DOCX)

S6 Table The R-square change after involving MIC-1/GDF15 serum levels in the regression analyses for Wave 1 MIC-1/GDF15 predicting two-year brain GM volume changes, adjusting for all other covariates (DOCX)

S7 Table The change in R-square after involving changes in MIC-1/GDF15 serum levels in the relationships between MIC-1/GDF15 serum level changes and brain GM changes, controlling for all other covariates (DOCX)
