# Supplementary material for: The Relationship of Serum Macrophage Inhibitory Cytokine – 1 Levels with Gray Matter Volumes in Community-Dwelling Older Individuals
Source: PLoS One. 2015 Apr 13;10(4):e0123399. doi: 10.1371/journal.pone.0123399 (PMC4395016; doi:10.1371/journal.pone.0123399)
Supplement: S1 Table — (DOCX) [file pone.0123399.s001.docx]

**S1 Table. Numbers of scans excluded for each structure after quality control**

|  | Cortical regions | Subcortical regions | | | | | | | |
| --- | --- | --- | --- | --- | --- | --- | --- | --- | --- |
|  |  | Hippocampus | Thalamus | Caudate | Putamen | Pallidum | Amygdala | Accumbens | Brainstem |
| Wave 1 | 20 | 16 | 11 | 15 | 20 | 19 | 20 | 20 | 12 |
| Wave 2 | 11 | 5 | 4 | 7 | 17 | 11 | 22 | 8 | 4 |
