# Supplementary material for: The Relationship of Serum Macrophage Inhibitory Cytokine – 1 Levels with Gray Matter Volumes in Community-Dwelling Older Individuals
Source: PLoS One. 2015 Apr 13;10(4):e0123399. doi: 10.1371/journal.pone.0123399 (PMC4395016; doi:10.1371/journal.pone.0123399)
Supplement: S2 Table — (DOCX) [file pone.0123399.s002.docx]

|  | | Wave 1 (n=274) | | | | Wave 2 (n = 155) | | | | |
| --- | --- | --- | --- | --- | --- | --- | --- | --- | --- | --- |
|  |  | Model 1 | | Model 2 | | Model 1 | | | Model 2 | |
|  |  | β | p | β | p | | β | p | β | p |
| Whole brain GM | | -0.147 | <0.001 | -0.147 | <0.001 | | -0.057 | 0.300 | -0.057 | 0.300 |
| Cortices | Total cortical GM | -0.122 | 0.003 | -0.115 | 0.005 | | -0.039 | 0.494 | -0.039 | 0.494 |
|  | Frontal | -0.094 | 0.041 | -0.094 | 0.041 | | -0.025 | 0.701 | -0.018 | 0.778 |
|  | Parietal | -0.090 | 0.050 | -0.090 | 0.050 | | -0.063 | 0.305 | -0.063 | 0.305 |
|  | Temporal | -0.134 | 0.003 | -0.134 | 0.003 | | -0.055 | 0.387 | -0.055 | 0.387 |
|  | Occipital | -0.168 | 0.002 | -0.132 | 0.016 | | 0.028 | 0.706 | 0.028 | 0.706 |
| Subcortical structures | Total subcortical GM | -0.156 | 0.002 | -0.160 | 0.002 | | -0.082 | 0.200 | -0.090 | 0.139 |
|  | Hippocampus | -0.118 | 0.058 | -0.118 | 0.058 | | -0.177 | 0.026 | -0.177 | 0.026 |
|  | Thalamus | -0.189 | 0.001 | -0.189 | 0.001 | | -0.097 | 0.229 | -0.106 | 0.177 |
|  | Caudate | -0.153 | 0.005 | -0.117 | 0.034 | | -0.033 | 0.666 | -0.033 | 0.666 |
|  | Putamen | -0.076 | 0.170 | -0.090 | 0.096 | | -0.058 | 0.498 | -0.062 | 0.468 |
|  | Pallidum | -0.140 | 0.031 | -0.140 | 0.031 | | -0.119 | 0.185 | -0.129 | 0.139 |
|  | Amygdala | -0.171 | 0.010 | -0.171 | 0.010 | | -0.032 | 0.722 | -0.032 | 0.722 |
|  | Accumbens | -0.213 | 0.001 | -0.213 | 0.001 | | -0.096 | 0.277 | -0.096 | 0.277 |
|  | Brainstem | -0.183 | <0.001 | -0.152 | 0.004 | | -0.044 | 0.542 | -0.045 | 0.526 |

**S2 Table. The association between MIC-1/GDF15 serum levels and brain GM volumes at Wave 1 and 2 in normal ageing participants**
