# Supplementary material for: The Relationship of Serum Macrophage Inhibitory Cytokine – 1 Levels with Gray Matter Volumes in Community-Dwelling Older Individuals
Source: PLoS One. 2015 Apr 13;10(4):e0123399. doi: 10.1371/journal.pone.0123399 (PMC4395016; doi:10.1371/journal.pone.0123399)
Supplement: S3 Table — (DOCX) [file pone.0123399.s003.docx]

**S3 Table. Regression analyses for the relationships of MIC-1/GDF15 at wave 1 with the changes in brain GM volume in normal ageing participants over two years**

| Prospective analyses (n = 119) | | | | | |
| --- | --- | --- | --- | --- | --- |
|  | | Model 1 | | Model 2 | |
|  |  | β | p | β | p |
| Whole brain GM | | 0.166 | 0.174 | 0.146 | 0.216 |
| Cortices | Total cortical GM | 0.124 | 0.306 | 0.105 | 0.371 |
|  | Frontal | 0.065 | 0.586 | 0.065 | 0.586 |
|  | Temporal | 0.191 | 0.132 | 0.170 | 0.165 |
|  | Parietal | 0.093 | 0.444 | 0.075 | 0.526 |
|  | Occipital | 0.105 | 0.401 | 0.105 | 0.401 |
| Subcortical structures | Total subcortical GM | 0.046 | 0.717 | 0.046 | 0.717 |
|  | Hippocampus | -0.076 | 0.558 | -0.076 | 0.558 |
|  | Thalamus | -0.080 | 0.537 | -0.080 | 0.537 |
|  | Caudate | 0.073 | 0.554 | 0.073 | 0.554 |
|  | Putamen | -0.071 | 0.583 | -0.071 | 0.583 |
|  | Pallidum | -0.042 | 0.744 | -0.042 | 0.744 |
|  | Amygdala | -0.027 | 0.839 | -0.027 | 0.839 |
|  | Accumbens | -0.210 | 0.099 | -0.210 | 0.099 |
|  | Brainstem | 0.014 | 0.914 | 0.014 | 0.914 |
