# Supplementary material for: The Relationship of Serum Macrophage Inhibitory Cytokine – 1 Levels with Gray Matter Volumes in Community-Dwelling Older Individuals
Source: PLoS One. 2015 Apr 13;10(4):e0123399. doi: 10.1371/journal.pone.0123399 (PMC4395016; doi:10.1371/journal.pone.0123399)
Supplement: S4 Table — (DOCX) [file pone.0123399.s004.docx]

**S4 Table. The association between MIC-1/GDF15 serum level changes and brain GM volumetric changes in two years in normal ageing participants**

| Longitudinal analyses (n = 119) | | | | | |
| --- | --- | --- | --- | --- | --- |
|  | | Model 1 | | Model 2 | |
|  |  | Beta | p | Beta | p |
| Whole brain GM | | -0.302 | 0.006 | -0.303 | 0.002 |
| Cortices | Total cortical GM | -0.213 | 0.054 | -0.217 | 0.026 |
|  | Frontal | -0.190 | 0.079 | -0.217 | 0.031 |
|  | Temporal | -0.224 | 0.053 | -0.224 | 0.030 |
|  | Parietal | -0.147 | 0.187 | -0.180 | 0.071 |
|  | Occipital | -0.168 | 0.144 | -0.168 | 0.144 |
| Subcortical structures | Total subcortical GM | -0.110 | 0.342 | -0.131 | 0.245 |
|  | Hippocampus | -0.038 | 0.751 | -0.038 | 0.751 |
|  | Thalamus | -0.220 | 0.060 | -0.220 | 0.060 |
|  | Caudate | 0.066 | 0.558 | 0.066 | 0.558 |
|  | Putamen | -0.090 | 0.460 | -0.090 | 0.460 |
|  | Pallidum | -0.155 | 0.201 | -0.155 | 0.201 |
|  | Amygdala | -0.148 | 0.228 | -0.148 | 0.228 |
|  | Accumbens | -0.153 | 0.198 | -0.153 | 0.198 |
|  | Brainstem | 0.012 | 0.918 | 0.012 | 0.918 |
