# Supplementary material for: The Relationship of Serum Macrophage Inhibitory Cytokine – 1 Levels with Gray Matter Volumes in Community-Dwelling Older Individuals
Source: PLoS One. 2015 Apr 13;10(4):e0123399. doi: 10.1371/journal.pone.0123399 (PMC4395016; doi:10.1371/journal.pone.0123399)
Supplement: S5 Table — (DOCX) [file pone.0123399.s005.docx]

S5 Table. The R-square change after involving MIC-1/GDF15 serum levels in the associations between MIC-1/GDF15 and brain GM volumes, controlling for all other covariates, at Wave 1 and 2

|  | Wave 1 | | Wave 2 | |
| --- | --- | --- | --- | --- |
|  | R^2^ change | p | R^2^ change | p |
| Whole brain GM | .014 | .000 | .004 | .094 |
| Total cortical GM | .010 | .001 | .002 | .217 |
| Frontal GM | .007 | .008 | .000 | .628 |
| Parietal GM | .010 | .001 | .002 | .207 |
| Temporal GM | .012 | .001 | .005 | .084 |
| Occipital GM | .006 | .038 | .000 | .716 |
| Insula GM | .003 | .031 | .007 | .018 |
| Total subcortical GM | .011 | .004 | .008 | .050 |
| Hippocampus GM | .013 | .008 | .010 | .062 |
| Thalamus GM | .025 | .000 | .010 | .057 |
| Caudate GM | .000 | .604 | .000 | .961 |
| Putamen GM | .001 | .380 | .000 | .722 |
| Pallidum GM | .015 | .008 | .001 | .507 |
| Amygdala GM | .002 | .353 | .005 | .233 |
| Accumbens GM | .035 | .000 | .009 | .081 |
| Brainstem GM | .012 | .002 | .003 | .225 |
