# Supplementary material for: The Relationship of Serum Macrophage Inhibitory Cytokine – 1 Levels with Gray Matter Volumes in Community-Dwelling Older Individuals
Source: PLoS One. 2015 Apr 13;10(4):e0123399. doi: 10.1371/journal.pone.0123399 (PMC4395016; doi:10.1371/journal.pone.0123399)
Supplement: S6 Table — (DOCX) [file pone.0123399.s006.docx]

**S6 Table. The R-square change after involving MIC-1/GDF15 serum levels in the regression analyses for Wave 1 MIC-1/GDF15 predicting two-year brain GM volume changes, adjusting for all other covariates**

|  | R^2^ change | p |
| --- | --- | --- |
| Whole brain GM | .011 | .102 |
| Total cortical GM | .010 | .102 |
| Frontal GM | .006 | .230 |
| Parietal GM | .011 | .097 |
| Temporal GM | .003 | .375 |
| Occipital GM | .002 | .551 |
| Insula GM | .015 | .060 |
| Total subcortical GM | .004 | .389 |
| Hippocampus GM | .001 | .573 |
| Thalamus GM | .002 | .539 |
| Caudate GM | .005 | .276 |
| Putamen GM | .000 | .867 |
| Pallidum GM | .003 | .456 |
| Amygdala GM | .000 | .852 |
| Accumbens GM | .003 | .403 |
| Brainstem GM | .003 | .392 |
