# Supplementary material for: The Relationship of Serum Macrophage Inhibitory Cytokine – 1 Levels with Gray Matter Volumes in Community-Dwelling Older Individuals
Source: PLoS One. 2015 Apr 13;10(4):e0123399. doi: 10.1371/journal.pone.0123399 (PMC4395016; doi:10.1371/journal.pone.0123399)
Supplement: S7 Table — (DOCX) [file pone.0123399.s007.docx]

**S7 Table. The change in R-square after involving changes in MIC-1/GDF15 serum levels in the relationships between MIC-1/GDF15 serum level changes and brain GM changes, controlling for all other covariates**

|  | R^2^ change | p |
| --- | --- | --- |
| Whole brain GM | .049 | .000 |
| Total cortical GM | .026 | .008 |
| Frontal GM | .015 | .051 |
| Parietal GM | .025 | .011 |
| Temporal GM | .018 | .042 |
| Occipital GM | .007 | .216 |
| Insula GM | .009 | .140 |
| Total subcortical GM | .052 | .002 |
| Hippocampus GM | .016 | .061 |
| Thalamus GM | .021 | .031 |
| Caudate GM | .005 | .271 |
| Putamen GM | .009 | .159 |
| Pallidum GM | .002 | .515 |
| Amygdala GM | .019 | .052 |
| Accumbens GM | .011 | .129 |
| Brainstem GM | .002 | .478 |
